# Identification of sex-linked markers in the sexually cryptic coco de mer: are males and females produced in equal proportions?

**DOI:** 10.1093/aobpla/plz079

**Published:** 2019-12-18

**Authors:** Emma J Morgan, Christopher N Kaiser-Bunbury, Peter J Edwards, Mathias Scharmann, Alex Widmer, Frauke Fleischer-Dogley, Chris J Kettle

**Affiliations:** 1 Department of Environmental Systems Science, ETH Zürich, Zürich, Switzerland; 2 Department of Biology, TU Darmstadt, Darmstadt, Germany; 3 Centre for Ecology and Conservation, College of Life and Environmental Sciences, University of Exeter, Cornwall Campus, Penryn, UK; 4 Singapore-ETH Centre, Singapore City, Singapore; 5 Department of Ecology and Evolution, University of Lausanne, Lausanne, Switzerland; 6 Seychelles Islands Foundation, Victoria, Mahé, Seychelles; 7 Bioversity International, Maccarese Rome, Italy

**Keywords:** Borasseae, conservation management, ddRAD sequencing, dioecy, *Lodoicea maldivica*, palm, reproductive ecology, sex-linked markers, sex ratios, Seychelles Islands

## Abstract

*Lodoicea maldivica* (coco de mer) is a long-lived dioecious palm in which male and female plants are visually indistinguishable when immature, only becoming sexually dimorphic as adults, which in natural forest can take as much as 50 years. Most adult populations in the Seychelles exhibit biased sex ratios, but it is unknown whether this is due to different proportions of male and female plants being produced or to differential mortality. In this study, we developed sex-linked markers in *Lodoicea* using ddRAD sequencing, enabling us to reliably determine the gender of immature individuals. We screened 589 immature individuals to explore sex ratios across life stages in *Lodoicea*. The two sex-specific markers resulted in the amplification of male-specific bands (Lm123977 at 405 bp and Lm435135 at 130 bp). Our study of four sub-populations of *Lodoicea* on the islands of Praslin and Curieuse revealed that the two sexes were produced in approximately equal numbers, with no significant deviation from a 1:1 ratio before the adult stage. We conclude that sex in *Lodoicea* is genetically determined, suggesting that *Lodoicea* has a chromosomal sex determination system in which males are the heterogametic sex (XY) and females are homogametic (XX). We discuss the potential causes for observed biased sex ratios in adult populations, and the implications of our results for the life history, ecology and conservation management of *Lodoicea*.

## Teaser

Our study explores sex ratios in the charismatic, dioecious palm *Lodoicea maldivica* (coco de mer), the largest seeded plant in the world. Males and females are visually indistinguishable when immature, only becoming sexually dimorphic as adults. We developed male-specific sex-linked markers using ddRAD sequencing, enabling us to determine the gender of immature individuals and explore how sex ratios change with environmental conditions and age. We conclude that dioecy in Lodoicea is genetically determined, and probably involves X/Y sex chromosomes. The two sexes are produced in equal numbers, and biased adult sex ratios are the result of differential mortality of mature plants.

## Introduction

Around 6 % of all flowering plants are dioecious, meaning that each individual is functionally either male or female (Renner 2014). Dioecy occurs in many families of flowering plants, and even within genera of otherwise monoecious species, indicating that the condition has evolved independently many times (Charlesworth 2002; Janousek and Mrackova 2010; Renner 2014) and in evolutionary terms is usually short-lived (Lewis 1942). Genetic mechanisms for dioecy vary widely, with one of the most common being the XY system (e.g. mammals)—in which an X chromosome, conferring recessive femaleness, pairs with a Y chromosome bearing the dominant genes associated with maleness. Other genetic mechanisms associated with dioecy are the ZW system (e.g. birds), in which the female is heterogametic, and multiple sex chromosome systems, in which the sex of an individual is determined by X/autosome ratios (e.g. in *Drosophila*, and *Rumex acetosa*; Parker and Clark 1991).

In theory, both the XY and ZW systems of sex determination should produce equal numbers of male and female progeny. However, biased sex ratios occur for a variety of ecological, genetic and physiological reasons that have been the subject of much research. One factor that may lead to bias is the intensity of pollination, with high intensities favouring female pollen (Stehlik and Barrett 2005; Stehlik *et al.* 2008). In this case, the reason is the accumulation of deleterious mutations in Y chromosomes as a result of low recombination, which reduces the vigour of ‘male’ pollen tubes, so that the successful pollen is more likely to be female (Muller 1964). Other genetic causes for biased sex ratios are inbreeding levels (Bailey and McCauley 2005; Barrett and Case 2006) and sex chromosome segregation distorters (Taylor and Ingvarsson 2003; Meiklejohn and Tao 2010). Ecological causes include variation in male tree density (Pickup and Barrett 2013), relative distances of seed and pollen dispersal (de Jong *et al.* 2002) and resource availability (Vandepitte *et al.* 2010; Adam *et al.* 2011).

Many long-lived dioecious plants, including several palms (Arecaceae), are economically or ecologically important (Henderson *et al.* 1995). For palms such as *Borassus flabellifer* and *Phoenix dactylifera,* which are commercially important for their fruits, female plants are clearly of more value than males. Being able to determine the sex of plants at an early stage avoids the need to grow large numbers of unproductive male plants. Recently, sex-determination methods using genetic markers have been developed, though some of these are inconvenient to use, poorly reproducible or costly (reviewed in Milewicz and Sawicki 2013).

This paper concerns the dioecious coco de mer palm, *Lodoicea maldivica* (J. F. Gmel.) Pers., which is an endemic palm of two small islands in the Seychelles, and a species of both economic and conservation importance. *Lodoicea* belongs to the tribe Borasseae (Arecaceae), which has a Gondwanaland distribution and in which dioecy is clearly an old-established trait (Dransfield *et al.* 2008). In its natural habitat, *Lodoicea* has a very prolonged immature phase, lasting as much as 50 years, during which it is impossible to distinguish between the sexes. Previous studies have shown that adult populations of *Lodoicea* often exhibit biased sex ratios, but it is unclear whether this bias is due to unequal numbers of males and females being ‘born’ or to differential rates of mortality subsequently (Fleischer-Dogley 2006).

We developed a novel molecular method that enabled us to determine the sex of immature *Lodoicea.* We applied this method to study sex ratios in sub-populations of *Lodoicea*, and investigate how these ratios change with age. We discuss the results in the context of the growing knowledge base on genetic and environmental factors influencing sex determination in dioecious plants, including the effects of parental pair relatedness, realised pollination distances and pollen availability, and we consider the implications for sustainable management of this emblematic species.

## Methods

### Study area and species

*Lodoicea maldivica* is endemic to the Seychelles (lat. 4°S, long. 55°E). Prior to the discovery of Praslin in 1743 (Bailey 1942), *Lodoicea* grew in dense monodominant stands that covered both Praslin (37 km^2^) and Curieuse (2.73 km^2^) (Brayer du Barre 1773, quoted in Fauvel 1909). However, due to exploitation for timber and the increasing demand from tourists for nuts, as well as several serious fires, the *Lodoicea* forests have recently become severely degraded, and there is now very little natural regeneration (Rist *et al.* 2010). The species is classified as endangered (Fleischer-Dogley *et al.* 2011) and the only remaining large populations are in the southern part of Praslin (where areas are still partially connected) and on Curieuse. In the north of Praslin and some areas on Curieuse, the species persists mainly as isolated individuals or in small patches, in scrubby habitat.

**Figure 1.**
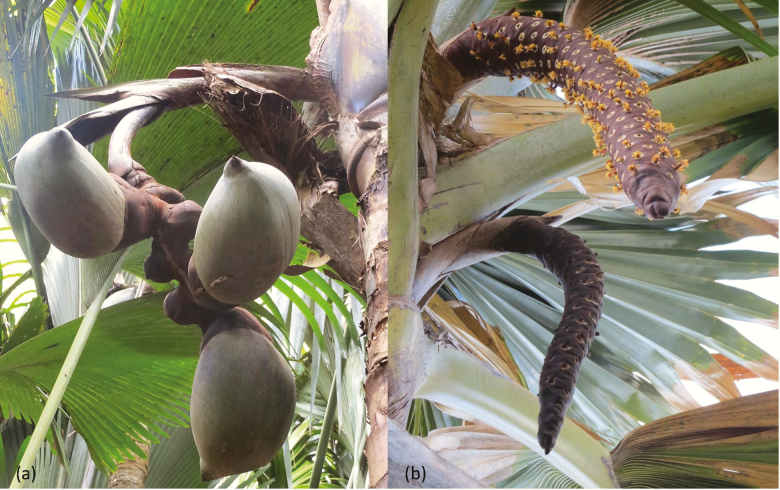
*Lodoicea maldivica* inflorescences. (A) Female with fruits and unfertilised flowers. (B) Male catkins.

The reproductive structures of the two sexes are very distinct (Fig. 1). Females produce inflorescences that bear between one and thirteen (Morgan *et al.* 2017a) of the largest flowers of any palm (~5 cm diameter), on a zigzag rachilla (Fig.1A). Male palms bear large catkins, 1.2- 1.8 m long (Corner 1966) and 8- 10 cm wide, consisting of 60–70 spirally arranged fragrant, yellow flowers embedded in leathery bracts (Fig.1B).

The seed (nut) of *Lodoicea* is the largest in the plant kingdom, with a mean fresh weight in the Vallée de Mai of 8.5 kg (Morgan *et al.* 2017a). Thanks to their unusual form, these seeds are highly sought after as souvenirs and fetch a price of €190- 450 each (Rist *et al.* 2010). Together, the sale of seeds and fees paid by visitors to the Vallée de Mai UNESCO World Heritage Site (Seychelles Islands Foundation 2009, unpubl. report) make a significant contribution to the Seychelles economy. Being able to identify female plants at seedling or juvenile stage would be useful both for conservation management and for the commercial production of nuts.

## Development of *Lodoicea* sex-linked markers

### Sequencing and bioinformatics

We identified potential sex-linked genetic markers using the approach of Scharmann *et al.* (2019). Genomic DNA was extracted from the leaf tissue of 20 male and 20 female trees, following an optimised version (Morgan *et al.* 2016) of the DNeasy® 96 Plant Kit (Qiagen, Hombrechtikon, Switzerland) manufacturer’s protocol. The genomes of the 40 individuals were sequenced using a ddRAD protocol (Peterson *et al.* 2012), with the restriction enzymes ecoRI and TaqI. The library with 40 individuals was sequenced in a single Illumina HiSeq 2500 lane for 136-bases single-end reads. Raw reads were de-multiplexed and quality-filtered (the entire read was discarded if quality dropped below Phred 20 in any window of 15 % read length) using process_radtags.pl from the Stacks pipeline (Catchen *et al.* 2013). Reads were mapped (following a customised dDocent-like pipeline; Puritz *et al.* 2014) against six different *de novo* reference assemblies, in order to reduce the chance of false positives caused by arbitrary choice of the assembly method. Using the reads of all 40 individuals, three alternative single-end references were assembled with identity cutoffs 0.8, 0.9 and 0.95 in clustering steps (vsearch, https://github.com/torognes/vsearch) and cd-hit-est (Li and Godzik 2006). Additionally, three alternative paired-end references were assembled (rainbow, Chong *et al.* 2012) using the reads of one male and one female sequenced on an Illumina MiSeq instrument (identical library protocol). This was done with reads trimmed to 146 bases to identity cutoffs 0.9 and 0.95 (cd-hit), and also with reads trimmed to 140 bases for identity cutoff 0.95. Single-end reads from the 40 individuals were mapped to each reference and formed the basis of the scan for male- or female-specific sequences.

Each individual was represented by an average of 2.9 million filtered reads (min. 370 000, max. 4.9 million), of which between 59 and 89 % (depending on the reference) could be mapped using bwa-mem (Li 2013) at a quality of ≥ 1. Presence/absence statistics (samtools, Li *et al.* 2009) for all individuals and reference contigs were then subjected to the privacy rarefaction procedure of Scharmann *et al.* (2019) (https://github.com/mscharmann/privacy-rarefaction). This algorithm separates real biological genomic presence/absence from stochasticity, yielding a list of contigs that, with high confidence, occur in one of the two sexes exclusively (with bootstrap support).

For all six *Lodoicea* reference assemblies, privacy rarefaction resulted in a divergence in counts of male- and female-specific candidates with increasing stringency, which is characteristic for organisms with sex chromosomes (Scharmann *et al.* 2019). More precisely, only male-specific candidate markers were obtained with high confidence, whereas apparent female-specific candidate markers were revealed as false-positives. This result is characteristic of a male-heterogametic system, and hence the existence of Y-chromosomes (Scharmann *et al.* 2019), although the Y-specific sequences were overall extremely rare. Consequently, we identified male-specific contigs suitable for a molecular sexing assay. From each reference assembly, we retained all contigs receiving at least 50 % bootstrap support for male specificity at stringency level ≥ 3. We enhanced the sequence length of these contigs by blasting for 100 % identical and full-length aligned matches in the unassembled (but quality filtered) 150-bases paired-end reads from the MiSeq run. The non-redundant candidate male-specific contigs (cd-hit-est at similarity 1.00) were ranked according to the cumulative support they received (sum of the passed stringency levels over all alternative reference assemblies), and the top 11 were used for primer design **[see Supporting Information—Text S1]**. [Detailed results for each scan can be found in **Supporting Information**—**Table S2** (read and mapping statistics per individual), **Supporting Information**—**Figure S2** (candidate contig figures) and **Supporting Information—Text S2** (list of contigs with > 50 % bootstrap support)].

### Molecular sexing assay

For a subset of eight samples of each sex, band sizes for each of the two male sex-linked markers were quantified via fragment analysis. PCR products (3 µL) from each primer pair were combined and added to 10 µL HIDI formamide and 0.15 µL GeneScan 500 LIZ Size Standard (Applied Biosystems). Samples were denatured for 3 min at 92 °C and then run on an ABI 3730xl automatic capillary sequencer (Applied Biosystems, Waltham, MA, USA). Electropherograms were scored with GeneMarker 2.6.0 (SoftGenetics, State College, PA, USA). Marker Lm123977 (contig ref_SE_cutoff10_vsearch0.8_cdhit0.8__123977_L131+PEcontig1_2103_21536_9724/1 [see **Supporting Information—Text S2**]) produced a strong peak (~ 9000 RFUs) at 405 bp only in the males, and another small peak (~ 400 RFUs) at 81 bp that amplified in males and females. Marker Lm435135 (contig ref_SE_cutoff10_vsearch0.95_cdhit0.95__435135_L131 [**see Supporting Information—Text S2**]) produced a peak (~ 8000 RFUs) in the males at 130 bp and a smaller peak (~ 300 RFUs) in both sexes (Table 1).

**Table 1.** Characteristics of the two male sex-linked loci in *Lodoicea maldivica*^a^. ^a^Values based on samples collected from eight males in the Vallée de Mai and Fond Peper, Praslin. ^b^Allele sizes include M13 tail (5′-TGTAAAACGACGGCCAGT-3′) attached to the forward primer (as described by Schuelke 2000).

Locus	Primer sequences (5’-3’)	Size (bp)^b^
Lm123977	F: GCCGGACCAACAAAATGTG	405
	R: CATTTACGATCCACACCAAAAGT	
Lm435135	F: TTCAAATATCAGCTTCACAAGTATTTT	130
	R: TTTCCAATCACTTTAGAAGACACG	

Gel electrophoresis using a 1.6 % agarose gel with a 100-bp ladder (both Promega) was used to assess the accuracy of the sex-linked markers **[see****Supporting Information—Figure S1]**. For this, we selected a subset of known female and male adults from across the geographic range. The adult samples (DNA extracted as above) were screened for the two sex-linked markers, along with negative controls and a positive control for each DNA sample (primer pair Lm2407, Morgan *et al.* 2016). No females amplified (*N* = 95 for both primers), while all but one of males amplified (Lm123977 *N* = 105; Lm435135 *N* = 98). The same male sample failed to amplify with both primer pairs. DNA samples of all *Lodoicea* offspring (see ‘Sample Collection and genotyping’ below) were screened using the two sex-linked markers, and positive and negative controls. Offspring sex assignments were determined with gel electrophoresis, and these were used for all future analyses.

## Exploring sex ratios in sub-populations of *Lodoicea maldivica* across age cohorts

### Sample collection and genotyping

**Figure 2.**
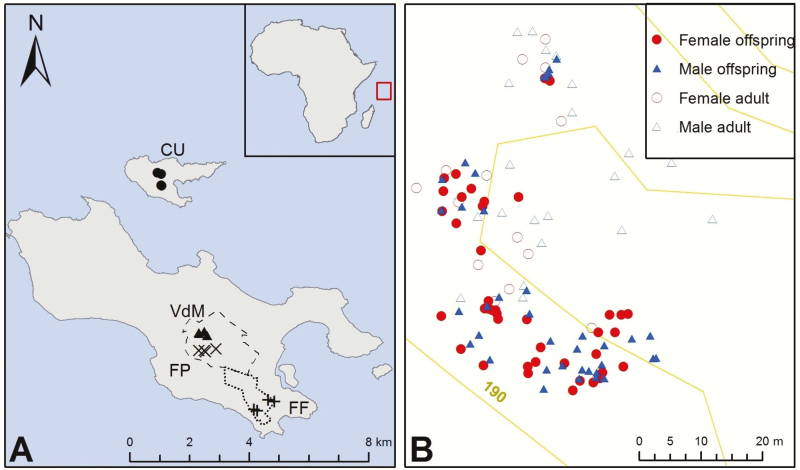
Locations of sampled *Lodoicea maldivica* on the Seychelles. (A) Centres of clusters on Praslin, indicated by: triangles = Vallée de Mai (VdM), 55°44′11ʺE, 4°19′43ʺS; cross = Fond Peper (FP), 55°44′ 17ʺE, 4°20′ 01ʺS; plus = Fond Ferdinand (FF), 55°203 45′ 39ʺE, 4°21′ 02ʺS, and circles = Curieuse (CU), 55°43′ 25ʺE, 4°16′ 45ʺS). The dashed and dotted lines indicate Praslin National Park and Ravin de Fond Ferdinand Nature Reserve, respectively. (B) Distribution of male and female offspring and adults in the VdM 3 and 4 clusters.

Leaf samples from across the whole of *Lodoicea*’s natural range (Fig. 2) were collected for DNA extraction. Plants were classified according to their growth stage: ‘adults’ were reproductive individuals producing flowers, ‘adolescents’ were non-reproductive individuals with a trunk, ‘juveniles’ were trunkless individuals with more than two leaves, ‘seedlings’ had one or two leaves, and ‘young seedlings’ were plants that had been weighed and measured as seeds in 2013, having been extracted from freshly fallen fruits, and allowed to germinate close to where they were found. We use the term ‘offspring’ to refer to all immature plants.

We sampled offspring from four naturally regenerating dense clusters of individuals showing minimal signs of human disturbance (Morgan *et al.* 2017b) within each of the four main sub-populations of *Lodoicea*: on Praslin (Vallée de Mai (VdM), Fond Peper (FP) and Fond Ferdinand (FF)), and on Curieuse (CU) (*N* = 493). Additionally, we sampled offspring from locations in the northern part of Praslin (Cherie Mon and Zimbabwe) and other areas outside of clusters in VdM (*N* = 48). We also sampled the bayonets (first leaf spikes) or first leaves of young seedlings in VdM and FP (*N* = 49) (total offspring *N* = 589), as well as leaf tissue from potential mother (*N* = 100) and father (*N* = 659) trees in the four sub-populations.

DNA extraction and genotyping of individuals at 12 polymorphic microsatellite loci were carried out as described by Morgan *et al.* (2016). We used genotype data for adults and offspring within clusters from Morgan *et al.* (2017b), and also genotyped additional offspring and young seedlings from outside the clusters [**see****Supporting Information—Table S1** for allele frequencies of additional samples]. Fragment analysis and scoring were carried out as in Morgan *et al.* (2016).

### Population surveys

A census of adult *Lodoicea* conducted in 2004 found that sex ratios in most populations in the native range were skewed/biased (data from Fleischer-Dogley (2006), presented in Table 2). We statistically tested deviation from a balanced sex ratio using an exact binomial test (two-sided) with the function stats::binom.test v. 3.1.2 (R Core Team 2014) in the RStudio environment v. 0.98.1102 (RStudio Team 2015). We also tested the effects of a number of ecological and genetic factors (see below) on the sex ratios of the offspring, in the same way (for female and male counts and total sample sizes for each group, see Table 3). We analysed whether the proportions of female offspring differed from the expected 0.5 within (i) each of the four sub-populations, (ii) each of the four clusters within each sub-population, (iii) each of the age cohorts (young seedlings, seedlings, juveniles and adolescent plants), and (iv) the entire sample.

**Table 2.** Surveyed *Lodoicea maldivica* sex ratios on Praslin and Curieuse. Offspring, and adult female, male and total counts, observed proportion of adult females (significant values in bold), probability that the observed adult sex ratios deviate from the expected 1:1 (with a confidence level of 95 %) are given. Complete census data from Fleischer-Dogley (2006).

Category	Offspring	Adult female	Adult male	Adult total	Prop. female	*P* (2-tailed)
**Population**						
Vallée de Mai	5624	623	818	1441	**0.432**	< 0.0001
Praslin NP	2823	653	428	1081	**0.604**	< 0.0001
Praslin–private land	1770	905	1031	1936	**0.467**	0.0045
Fond Ferdinand	4528	675	705	1380	0.489	0.44
Curieuse	2043	948	802	1750	**0.542**	0.0005

**Table 3.** Analysed *Lodoicea maldivica* offspring sex ratios on Praslin and Curieuse. Female, male and total counts, observed proportion of females (significant values in bold), probability that the observed sex ratios deviate from the expected 1:1 (with a confidence level of 95 %) are given. Categories include total offspring, and offspring: from the four main sub-populations, within each of the age cohorts (across all populations), produced from relatively shorter (≤ 21.9 m) and longer distance (> 21.9 m) pollination events, with relatively low and high relatedness (*F*) values of the assigned parents, and offspring assigned to two individual females that produced an excess of females. *F* = kinship coefficient (Loiselle *et al.* 1995).

Category	Female	Male	Total	Prop. female	*P* (2-tailed)
**All offspring**	309	280	589	0.525	0.25
**Sub-population**					
Vallée de Mai	112	110	222	0.505	0.95
Fond Peper	77	66	143	0.538	0.40
Fond Ferdinand	64	59	123	0.520	0.72
Curieuse	53	40	93	0.570	0.21
**Cohort**					
Young seedling	23	26	49	0.469	0.78
Seedling	28	26	54	0.519	0.89
Juvenile	247	213	460	0.537	0.12
Adolescent	11	15	26	0.423	0.56
**Pollen flow distance (m)**					
≤ 21.9	40	30	70	0.571	0.28
> 21.9	34	35	69	0.493	1.00
**Parent kinship (*F*)**					
≤ 0.068	35	35	70	0.500	1.00
> 0.068	39	30	69	0.565	0.34
**Offspring from individual females**					
Female 1—Vallée de Mai	11	2	13	**0.846**	0.02
Female 2—Curieuse	10	2	12	**0.833**	0.04

### Factors influencing offspring sex

To analyse the effect of pollen flow distance on the sex of offspring, we used data from a maternity analysis from Morgan *et al.* (2017b). Paternity analysis was determined by the Δ-estimated statistic, using 10 000 simulations, 0.01 error rate at the loci and 0.961 loci typed, using the software CERVUS 3.0 (Marshall *et al.* 1998; Kalinowski *et al.* 2007). We simulated 0.05 candidate fathers that are related to offspring at 0.02. All adult males sampled within a radius of between 80 and 120m from assigned mother trees were considered as potential fathers (assuming 0.5 pollen donors sampled). The strict confidence level of 95 % for the trio assignment (mother–father–offspring) was used. Combined exclusion probabilities for the first and second parents were 0.9961 and 0.9999, respectively.

Realised pollen dispersal distances for each offspring were calculated as the Euclidian distances between the assigned mother and father trees, and we divided offspring into two groups according with pollination events (i.e. assigned parents) less than and greater than the overall median level (≤ 21.9 or > 21.9 m).

We also tested directly whether parental relatedness had any influence on the sex of the offspring. For this, we calculated the pairwise kinship coefficient (*F*; Loiselle *et al.* 1995) for each parent-pair (SPAGeDI 1.4c; Hardy and Vekemans 2002), using the whole adult plant dataset as the reference sample. We then divided the offspring into two groups according with parental relatedness less than and greater than the overall median level (*F ≤* 0.068 or > 0.068).

We also tested whether the proportions of female offspring produced by individual female palms deviated from the expected level. We used maternity assignments to match offspring to mother trees, to test sex production at the finest scale (Morgan *et al.* 2017b).

As estimates of pollen availability, we recorded the distance of the mother tree to the nearest adult male, and the number of males within a 10 m radius of the mother. The correlation between each of these measurements and the proportion of female offspring assigned to each mother tree (number of offspring assigned to each mother ranged from 1 to 30, mean = 4.6), was tested using a Pearson’s correlation with the R package stats::cor.test (R Core Team 2014).

Finally, for a sample of 49 seeds planted in 2013, we tested for any relationship between seed size (fresh weight and length) and the sex of the seedling.

## Results

### Sex ratios of adults and offspring

Both of the markers developed in this study amplified a male-specific band (Lm123977 at 405 bp and Lm435135 at 130 bp), allowing us to reliably determine the gender of immature *Lodoicea* plants. Previous surveys had shown highly biased sex ratios in three of the four adult sub-populations: Praslin National Park (which includes Fond Peper plus the wider area) and Curieuse had strong female biases (0.604 and 0.542 female; binomial test *P* < 0.0001 and *P* = 0.0005, respectively), whereas Vallée de Mai and private land on Praslin had significant male biases (0.432 and 0.467 female; binomial test *P* < 0.0001 and *P* = 0.0045, respectively). Only the Fond Ferdinand sub-population showed not significant sex bias.

In contrast, no significant deviation from a 1:1 sex ratio was detected in the entire sample (0.525; Table 3), or within any of the four sub-populations (female ratio range 0.505- 0.570; Table 3) (binomial test all *P* > 0.05). Equal sex ratios were also observed in all age cohorts of offspring from young seedlings to adolescent trees (female ratios between 0.423 and 0.537, binomial test all *P* > 0.05). Two female palms, one in VdM and one on CU, did produce offspring with a significant female-bias (11/13 and 10/12 females, respectively; binomial test both *P* < 0.05; Table 3), yet no apparent reason for this bias could be determined.

### Effects of pollen flow distance, genetic relatedness of parent-pairs and male tree density on offspring sex ratios

Realised pollination distances were not related to female-biased sex ratio in adult population (Table 3; all *P* > 0.05). The range in kinship (*F*, Loiselle *et al.* 1995) of parent-pairs of female offspring ranged from -0.145 to 0.409, and in males from -0.192 to 0.392, but the level of relatedness of the parent trees also had no effect on the sex of the resultant offspring (binomial test both *P* > 0.05).

Pearson’s correlation coefficients were calculated to investigate possible relationships between the proportion of female offspring produced by mother trees and two measures of pollen availability (distance to the nearest male, which ranged from 0.9 to 18.6 m, and number of males within 10 m, which ranged from 0 to 8). However, neither measure yielded a significant relationship (*r*_56_ = 0.252, *P* = 0.06 for distance; *r*_56_ = -0.177, *P* = 0.18 for number of males; **[see****Supporting Information—Figure S3****]**).

Female seeds ranged in fresh weight from 6 to 12 kg, with a mean and SE of 8.9 ± 0.4 kg (*N* = 23). Male seeds ranged in weight from 3 to 13 kg, with a mean and SE of 8.4 ± 0.5 kg (*N* = 26; **[see****Supporting Information—Figure S4****]**). The average lengths of female and male seeds were 30.8 (± 0.5) cm and 30.3 (± 0.7) cm, respectively **[see****Supporting Information—Figure S4** for details]. We found no relationship between the mass or length of seeds and the sex of the plant (Pearson’s correlation, both *P* > 0.05).

## Discussion

The sex-specific molecular markers for *Lodoicea maldivica* described here are among the first to be developed for any palm species (but see George and Karun 2011 and Cherif *et al.* 2013). Using these markers, we were able to determine reliably the sex of immature plants of *Lodoicea* sampled from across its native range on the islands of Praslin and Curieuse. Although sex ratios of most adult populations were strongly skewed, we detected no departure from a 1:1 ratio in the offspring. We found no environmental effects on sex ratios of immature plants, such as density of male plants and distance to the nearest male. Based upon these results and other available information, we can draw several conclusions concerning the nature of dioecy and the factors influencing sex ratios in *Lodoicea*.

### 

#### Dioecy in Lodoicea has a genetic basis and probably involves X/Y sex chromosomes.

The discovery and PCR-validation of male-specific markers but not of female-specific markers suggests the existence of X/Y sex chromosomes. Cytological studies have shown that *Lodoicea* has 2n = 34, 36 chromosomes (Sharma 1970; Dransfield *et al.* 2008), but no information is available about the nature of the sex chromosomes. Thus, it is not known whether the X- and Y-chromosomes are homomorphic, as is the case in many plant species, or heteromorphic, as in the genera *Silene*, *Rumex* and *Cannabis* (Westergaard 1958; Parker 1990). In our smallest and largest ddRAD assemblies, in terms of number of total markers, the incidence of male-specific markers when 10 males and 10 females were compared was 5.29/94 968 (0.0056 %) and 0.59/38 830 (0.0015 %), respectively (**see****Supporting Information—Figure S2**). These proportions of male-specific markers are considerably lower than those detected for other dioecious plants, whether with heteromorphic (*Silene latifolia*: 1.5 %) or homomorphic (*Nepenthes*: 0.02–0.1 %; Scharmann *et al.* 2019) sex chromosomes, leading us to conclude that *Lodoicea* sex chromosomes are most probably homomorphic. However, given that we do not know the genome size of *Lodoicea*, the small proportion of male-specific markers could still represent a physically large Y-linked region.

Although information on genome size of *Lodoicea* is not available, estimates from some of its closest relatives in the tribe Borasseae predict a large genome (*Bismarkia nobilis:* 1n = 36, 1C = 2 Gb; *Borassus flabellifer*: 1n = 36, 1C = 8.6 Gb; *Latania lontaroides*: 1n = 28, 1C = 3.5 Gb, Leitch *et al.* 2019). Since all borassoid palms are strictly dioecious (Dransfield *et al.* 2008), it is possible that the Y-chromosomal system of *Lodoicea* is ancestral in this group. Dioecy has evolved multiple times independently within the palm family, including in the date palm genus, *Phoenix* (Torres *et al.* 2018). We suggest that X/Y sex chromosomes in *Lodoicea* and Borasseae in general are similar to those found in the date palms (Cherif *et al*. 2013, 2016; Mathew *et al.* 2014; Maryam *et al.* 2016), in the sense that they are homomorphic, highly conserved and shared among a larger group of species, although date palms and Borasseae represent two unrelated evolutionary origins of dioecy (Nadot *et al.* 2016). Further studies on the *Lodoicea* karyotype and genome, as well as on other genera in the tribe Borasseae, would be useful as a starting point for understanding the evolution of these sex chromosomes.

#### *The two sexes of Lodoicea are* ‘born’ *in approximately equal numbers.*

If the sex of offspring is genetically determined through an X/Y chromosome system, we would expect there to be equal numbers of male and female offspring produced, as is the case in the date palm, *Phoenix dactylifera* (Saadi 1990). However, various processes operating both pre- and post-zygotically may distort the balanced sex ratio expected from theory.

One prezygotic effect known as certation, occurs through male and female pollen tubes growing at different rates on the stigma (Correns 1922). This effect can lead to a biased sex ratio, especially when because of high pollen availability there is strong competition between male and female pollen. Very often the male pollen is at a disadvantage because of negative mutations that accumulate in the sex-determining region of the Y chromosome. The fact that we found no influence of pollen availability (measured in terms of numbers of male trees and distance to the nearest male) upon the sex ratio of progeny suggests that this source of bias is unimportant in *Lodoicea*. This, in turn, is consistent with dioecy in *Lodoicea* being a long established, stable system from which negative mutations have been purged.

The degree of inbreeding may also influence sex determination in dioecious species (Bailey and McCauley 2005; Barrett and Case 2006). For example, a female bias may develop when both pollen and seed dispersal are limited, which tends to increase the inbred population’s relative contribution to the next generation. On the other hand, when seed dispersal is much more limited than pollen dispersal, which appears to be the case for *Lodoicea*, a male bias would reduce the chance of related individuals being close together and competing for the same resources (de Jong *et al.* 2002). *Lodoicea* across all sub-populations are relatively highly inbred with short-distance seed dispersal (Morgan *et al.* 2017b) and relatively short pollen dispersal (unpubl. data). Our results provide no evidence for such inbreeding effects, despite the fact that natural sub-populations of *Lodoicea* show high levels of inbreeding associated with an intense fine-scale spatial genetic structure (Morgan *et al.* 2017b).

These negative results all point in the same direction—in *Lodoicea*, the two sexes are produced in equal numbers. Similarly, almost equal sex ratios have also been found in other dioecious tropical palms, including *Chamaedorea tepejilote* (Oyama 1990) and *C. pinnatifrons* (Ataroff and Schwarzkopf 1992; as *C. bartlingiana*), *Mauritia flexuosa* (Urrego Giraldo 1987) and *Phytelephas seemannii* (Bernal 1998). The only contradictory results in our study were for two female plants that produced almost exclusively female individuals. The reasons for this are unknown, but could reflect some genetic incompatibility between those particular maternal genotypes and the male sex-determining region of the Y-chromosome, or a cryptic sex-ratio meiotic driver acting, such as those found in *Drosophila* (Sturtevant and Dobzhansky 1936).

#### The seeds of Lodoicea are not sexually dimorphic.

Sexual dimorphism in seeds is known to occur in some species, notably *Spinacia oleracea* (Freeman *et al.* 1994) and *Rumex nivalis* (Stehlik and Barrett 2005). Given the high metabolic cost of producing the huge seeds of *Lodoicea*, it would perhaps not be surprising if female seeds were supplied with more food reserves (Cipollini 2013). Such a dimorphism might lead to more male seeds being produced, especially under conditions of low nutrient availability (Freeman *et al.* 1981), which would also reduce parent–offspring conflict (Trivers 1974). We found no evidence for such effects; however, male seeds did vary more than female seeds in length and mass, but the mean values of these parameters did not differ significantly between the sexes.

#### Biased sex ratios in adult populations are the result of differential mortality of mature plants.

Immature cohorts of *Lodoicea* did not depart significantly from a 1:1 sex ratio at any developmental stage or in any sub-population (although it should be noted that one male adult palm failed to amplify using our primers during screening). In contrast, sex ratios in adult populations did deviate significantly from parity, though not consistently in the same direction. Whereas, the Vallée de Mai and Praslin private land had more males than females, Fond Ferdinand and Praslin National Park had more females. One reason for this discrepancy is certainly human interference, which has resulted in skewed sex ratios in other long-lived plants and animals (Clutton-Brock and Lonergan 1994; Holand *et al.* 2003; Horn *et al.* 2012). None of the populations in which *Lodoicea* grows is in a natural condition. Unprotected sites such as state-owned lands on Praslin may have been subjected to higher levels of selective felling of one sex over the other: either felling of females for their reputedly superior wood (Edwards *et al.* 2002), or preserving females for the valuable nuts they produce.

The population structure at FF, the site with the least biased sex ratio, is probably closer to the natural condition than elsewhere, because this site has been protected for longer. In a survey of the VdM conducted in 1976, Savage and Ashton (1983) found similar numbers of young male and female palms entering maturity, but that the proportion of females declined with increasing height (and therefore age); the tallest male trees in their sample were 28 m high, while the tallest females were only 18 m. Historical accounts from the 19th century also describe male trees reaching greater heights than female trees, with one report of two male trees exceeding 50 m in height (Fauvel 1915). Savage and Ashton (1983) and Silvertown (1987) attributed the shorter lifespans of female trees to the large weight of fruits (in some cases amounting to several hundred kilograms), which made them more vulnerable to high winds. However, this explanation seems unlikely, since fallen *Lodoicea* are very rare, with most trees, both male and female, dying ‘on their feet’.

A more probable explanation for the earlier death of females is the natural pattern of regeneration in *Lodoicea* forest. Because fruits have no mechanism for dispersal apart from gravity, young plants grow up in dense clusters close to the mother trees (Morgan *et al.* 2017b). As the juveniles develop, they produce huge leaves on enormously elongated petioles, which in due course overtop the mother, resulting in intense competition between mother and progeny.

### Implications for management

Because they develop slowly, the majority of plants in most *Lodoicea* populations are immature individuals (e.g. 80 % of individuals in VdM are immature; Fleischer Dogley *et al.* 2011), whose sex could not previously be determined. Our results demonstrate that these immature cohorts have balanced sex ratios, which bodes well for the future of these populations and for maintaining genetic diversity. It is also important from a management perspective that the seeds do not appear to be sexually dimorphic, because it suggests that the current practice of using smaller, misshaped seeds for forest regeneration, while selling the larger, better shaped seeds, will not affect the sex ratios of populations in the future. It would be worth repeating this study with a larger sample; however, to confirm our provisional conclusion that seeds are not sexually dimorphic.

*Lodoicea* nuts attract high prices and are a desirable commodity. They could potentially be grown commercially, which as well as increasing revenue might reduce the poaching of nuts from protected areas, enabling *ex situ* conservation and supporting the selection of balanced sex ratios of populations in botanic gardens. If commercial production were to be established, the method for sex determination described here would be very useful for setting out plantations of predominantly female plants. In addition, our molecular sexing might be applicable to other species within the tribe Borasseae, notably the economically important *Borassus flabellifer*.

## Supporting Information

The following additional information is available in the online version of this article—

**Figure S1.** Digital photograph of agarose gel after electrophoresis of female and male *Lodoicea maldivica* PCR products, amplified with the Lm123977 marker.

**Figure S2.** Privacy rarefaction plots showing evidence for male-specific (Y-hemizygous) contigs in *Lodoicea,* based on six alternative reference assemblies.

**Figure S3.** Proportion of female offspring produced by each *Lodoicea maldivica* mother tree (*N* = 58), in relation to: (a) the isolation distance of their mother tree to the nearest male *Lodoicea* (regression line of the non-significant relationship; *r* = 0.252, *P* = 0.06); and (b) the number of male *Lodoicea* within a 10 m radius of their mother tree (regression line of the non-significant relationship; *r* = -0.177, *P* = 0.18).

**Figure S4.** Boxplots showing (a) masses (kg) and (b) lengths (cm), of female and male *Lodoicea maldivica* seeds (*N* = 49).

**Table S1.** Genetic properties of 12 microsatellite markers in *Lodoicea maldivica* offspring.

**Table S2.** Read and mapping statistics per individual (20 females and 20 males) for six alternative *de novo* reference assemblies.

**Text S1.** Sex-linked marker design for *Lodoicea maldivica.*

**Text S2.** Candidate male-specific contigs obtained from privacy rarefaction, and screened during development of molecular sexing primers.

plz079_suppl_Supplementary_Figures

## Data

Demultiplexed Illumina sequencing data (ddRAD) produced for this study were deposited at the European Nucleotide Archive ENA (https://www.ebi.ac.uk/ena) under accession number PRJEB34653. Remaining data are included as Supporting Information.

## Sources of Funding

This research was funded by Eidgenössische Technische Hochschule Zürich (ETH-37 12-1). C.N.K-B. received funding from the Deutsche Forschungsgemeinschaft (KA 3349/2-1 and KA 3340/3-1). Support was provided by the Rübel Foundation.

## Contributions by the authors

E.J.M, C.N.K-B., P.J.E. and C.J.K. conceived the ideas and designed the methodology. F.F-D. made fieldwork possible. E.J.M. conducted the field and laboratory work. C.N.K-B., P.J.E., F.F-D. and C.J.K provided advice and input to field work. C.N.K-B., P.J.E. and C.J.K. supervised data analysis. M.S. designed and conducted bioinformatics analyses. A.W. provided advice and made marker design possible. E.J.M wrote the initial draft of the manuscript. E.J.M., C.N.K-B., P.J.E. and C.J.K reviewed and edited subsequent versions of the manuscript, with contributions from M.S. and A.W. All authors gave final approval for publication.

## Notes

Evolution & Diversity. Chief Editor: Jeremy Beaulieu
