# Supplementary material for: Identification of sex-linked markers in the sexually cryptic coco de mer: are males and females produced in equal proportions?
Source: AoB Plants. 2019 Dec 18;12(1):plz079. doi: 10.1093/aobpla/plz079 (PMC6964228; doi:10.1093/aobpla/plz079)
Supplement: plz079_suppl_Supplementary_Figures [file plz079_suppl_supplementary_figures.pdf]

Supporting information

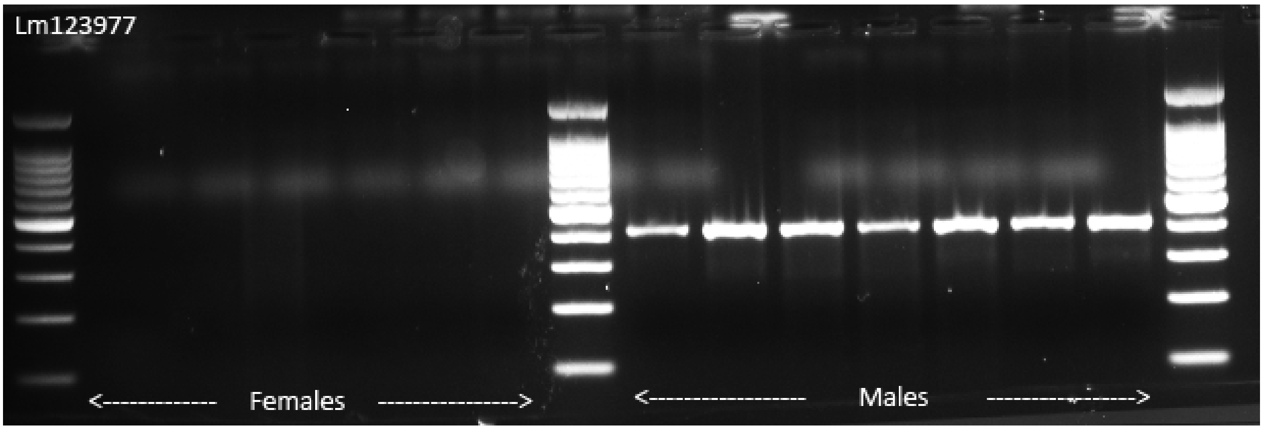

Fig S1. Digital photograph of agarose gel after electrophoresis of female and male *Lodoicea maldivica* PCR products, amplified with the Lm123977 marker. DNA was stained with florescent GelRed™ (Biotium, Fremont, California, USA). 100 bp ladders shown.

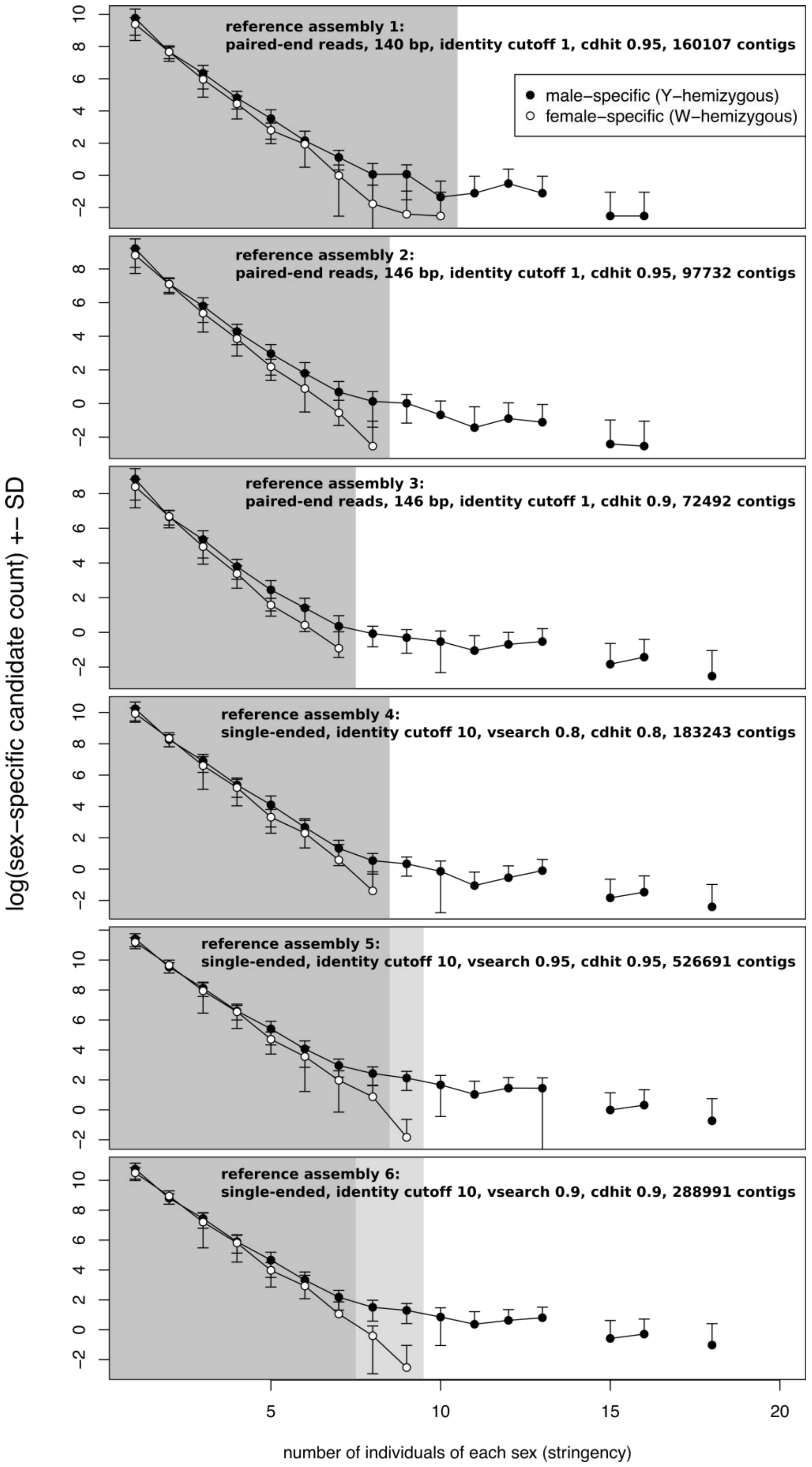
reference assembly 1:
paired-end reads, 140 bp, identity cutoff 1, cdhit 0.95, 160107 contigs
male−specific (Y−hemizygous)
female−specific (W−hemizygous)
reference assembly 2:
paired-end reads, 146 bp, identity cutoff 1, cdhit 0.95, 97732 contigs
reference assembly 3:
paired-end reads, 146 bp, identity cutoff 1, cdhit 0.9, 72492 contigs
reference assembly 4:
single-ended, identity cutoff 10, vsearch 0.8, cdhit 0.8, 183243 contigs
reference assembly 5:
single-ended, identity cutoff 10, vsearch 0.95, cdhit 0.95, 526691 contigs
reference assembly 6:
single-ended, identity cutoff 10, vsearch 0.9, cdhit 0.9, 288991 contigs
log(sex−specific candidate count) +− SD
number of individuals of each sex (stringency)

Fig S2. Privacy rarefaction plots showing evidence for male-specific (Y-hemizygous) contigs in *Lodoicea*, based on six alternative reference assemblies. Counts of sex-specific contigs are shown on the y-axis (natural logarithm scale) as a function of the number of individuals of each sex sampled to score sex-specificity, on the x-axis. If sequencing reads from only one of the sexes can be aligned, the contig is scored as sex-specific. Dots represent averages, and whiskers one standard deviation of 200 bootstrapped combinations of males and females. Dark grey background shading: no difference between the sexes in candidate contig counts, light grey shading: significant differences, white background: only one sex has sex-specific candidates (here, the males). Note that even though 20 males and 20 females were sequenced, none of the later PCR-validated male-specific markers was present in the sequencing data of all 20 males (not even in 19). Due to such noisy, stochastic absence of sequencing reads, the largest number of analysed individuals (stringency) at which a male-specific contig was obtained was 18.

(a)

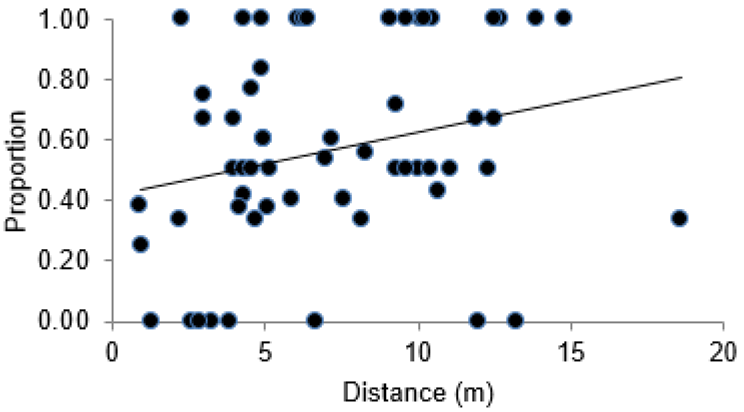

(b)

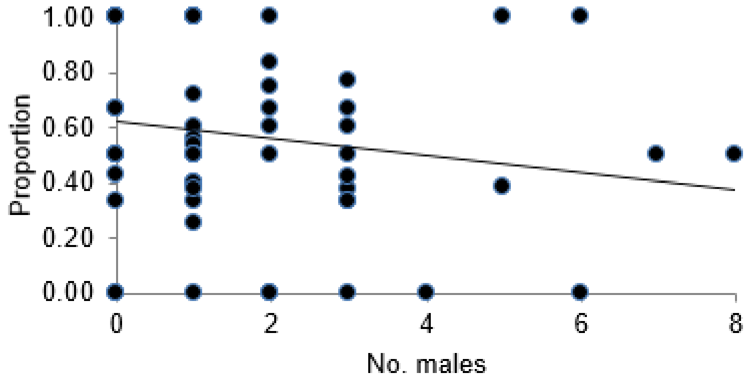

Fig S3. Proportion of female offspring produced by each *Lodoicea maldivica* mother tree (*N* = 58), in relation to: (a) the isolation distance of their mother tree to the nearest male *Lodoicea* (regression line of the non-significant relationship; *r* = 0.252, *P* = 0.06); and (b) the number of male *Lodoicea* within a 10 m radius of their mother tree (regression line of the non-significant relationship; *r* = -0.177, *P* = 0.18). Established offspring were assigned to mother trees using maternity analyses. Total offspring sample size *N* = 266. Numbers of offspring assigned to each mother tree ranged from one to 30.

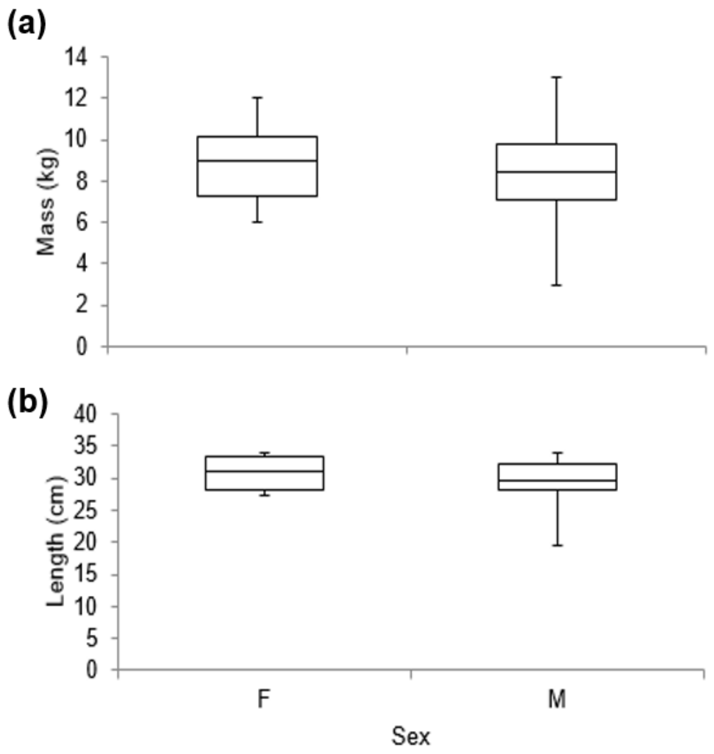

Fig S4. Boxplots showing (a) masses (kg) and (b) lengths (cm), of female and male *Lodoicea maldivica* seeds (*N* = 49). First quartiles, medians and third quartiles indicated by boxes. Whiskers extend to minimum and maximum values. Seeds were weighed and measured in 2013 and sexed with two sex-linked markers (Lm123977 and Lm435135) using DNA extracted from the first bayonet or leaf, in 2015.

Table S1. Genetic properties of 12 microsatellite markers in *Lodoicea maldivica* offspring. Offspring outside clusters were seedlings, juveniles and adolescent trees sampled in Cherie Mon, Zimbabwe, Vallée de Mai or Fond Ferdinand, Praslin island. Young seedlings grew from seeds left to regenerate in 2013. *N*, sample size; N_a, number of alleles; uH_E, unbiased expected heterozygosity; H_O, observed heterozygosity; *HWE*, Hardy-Weinberg Equilibrium.

[a] Deviations from *HWE* using χ^2 tests: $*P \leq 0.05$, $** P \leq 0.01$, $*** P \leq 0.001$; ns = not significant.

	Offspring outside clusters *(N = 48)*				Young seedlings (*N* = 49)			
Locus	N_a	H_O	uH_E	*HWE*[a]	N_a	H_O	uH_E	*HWE*[a]
Lm4716	3	0.617	0.502	3.262 ns	5	0.542	0.578	8.551 ns
Lm2630	14	0.689	0.868	95.163 ns	13	0.422	0.812	277.124***
Lm8853	4	0.375	0.576	43.639***	4	0.375	0.508	7.561 ns
Lm5648	12	0.729	0.851	96.736**	9	0.809	0.837	122.064***
Lm6782	15	0.370	0.703	239.636***	12	0.489	0.795	133.116***
Lm1153	14	0.587	0.841	151.860***	11	0.556	0.815	115.056***
Lm4293	5	0.186	0.360	42.878***	6	0.217	0.387	72.077***
Lm1750	5	0.660	0.703	14.785 ns	5	0.612	0.659	8.119 ns
Lm2407	7	0.354	0.483	30.703 ns	5	0.347	0.322	1.909 ns
Lm6026	9	0.479	0.798	177.922***	8	0.375	0.709	107.784***

Lm0144	8	0.500	0.739	34.741 ns	6	0.449	0.651	0.010*
Lm2071	12	0.771	0.856	66.680 ns	12	0.857	0.856	57.087 ns

[a] Deviations from *HWE* using χ^2 tests: **P* ≤ 0.05, ** *P* ≤ 0.01, *** *P* ≤ 0.001; ns = not significant.

Table S2. Read and mapping statistics per individual (20 females and 20 males) for six alternative *de novo* reference assemblies.

Sample	Filtered read count	Reference assembly					
		1	2	3	4	5	6
F1	2476602	1742452	1496518	1495208	1458899	2205327	2017362
F2	2447404	1714600	1461449	1461593	1462391	2182864	2008812
F3	3356752	2349691	2024555	2026204	1966710	2981769	2728661
F4	3082747	2155978	1846900	1845225	1849114	2744832	2522469
F5	3099330	2166579	1855537	1856907	1852730	2763265	2541487
F6	2038683	1431341	1227881	1228583	1217966	1815709	1673603
F7	3797833	2681836	2296112	2291038	2266891	3381520	3104403
F8	2429562	1713557	1468308	1468107	1442304	2165950	1982977
F9	370922	261164	223435	222737	218816	331906	302866

F10	2128985	1504849	1283097	1279195	1252163	1895301	1730624
F11	2677562	1875109	1601643	1601582	1605167	2385779	2198647
F12	4476654	3130071	2690421	2692295	2648144	3991590	3660343
F13	1121131	799081	681067	678030	662583	997499	912886
F14	3224552	2242422	1918703	1920681	1944280	2873491	2651101
F15	3201770	2264541	1931254	1925185	1909285	2854465	2622567
F16	3170988	2214569	1898622	1898829	1912693	2820464	2600877
F17	3151360	2201010	1882981	1886120	1860503	2809773	2573482
F18	3434662	2448960	2087232	2071563	2071370	3063713	2799830
F19	1485113	1040139	885385	885349	895960	1324080	1223132
F20	1574339	1112673	946271	940365	966312	1406887	1296666
M1	1433183	1007389	859724	858869	860424	1282351	1179070
M2	3296751	2299743	1967688	1970369	1975195	2937894	2706949
M3	3763510	2630527	2252847	2251743	2250702	3357997	3091159
M4	3427846	2430514	2071636	2059117	2088943	3054998	2798565
M5	3602321	2583782	2197253	2176128	2193295	3219623	2943434
M6	2972387	2110124	1802108	1791234	1810769	2645235	2426092
M7	4039043	2852565	2445599	2442026	2390544	3601943	3298448

M8	3512477	2496022	2147697	2141435	2043700	3121800	2840489
M9	4818225	3395486	2915937	2909838	2854129	4303317	3928164
M10	4625543	3268403	2793387	2788401	2765073	4126078	3778205
M11	3700688	2586735	2215075	2215720	2197924	3304534	3035541
M12	2348873	1678660	1435642	1420499	1420308	2079968	1906279
M13	4899753	3438167	2954408	2946673	2896255	4352419	3974563
M14	3100316	2163107	1857557	1856842	1826552	2752332	2519368
M15	2135044	1501993	1285420	1283108	1264989	1901541	1742187
M16	4392027	3090940	2653131	2649018	2579345	3901876	3569682
M17	2198362	1547935	1328625	1328302	1293266	1956195	1788918
M18	1009794	708611	607062	606179	601489	899634	828023
M19	1999191	1407799	1211054	1207497	1185128	1776555	1629230
M20	1981524	1389555	1191109	1191270	1173101	1764300	1619041

Note: Reference assemblies 1: paired-end reads, 140 bp, identity cutoff 1, cdhit 0.95, 160107 contigs; 2: paired-end reads, 146 bp, identity cutoff 1, cdhit 0.95, 97732 contigs; 3: paired-end reads, 146 bp, identity cutoff 1, cdhit 0.9, 72492 contigs; 4: single-ended, identity cutoff 10, vsearch 0.8, cdhit 0.8, 183243 contigs; 5: single-ended, identity cutoff 10, vsearch 0.95, cdhit 0.95, 526691 contigs; 6: single-ended, identity cutoff 10, vsearch 0.9, cdhit 0.9, 288991 contigs.

Text S1. Sex-linked marker design for *Lodoicea maldivica.*

Male sex-linked nuclear markers were designed for *Lodoicea* using Primer3web 4.0.0 (Koressaar & Remm 2007; Untergasser *et al.* 2012). We tested 11 potentially promising primer pairs with PCRs using eight males and eight females, and the most reliable two pairs (Table 1) that amplified only in males, were optimised. PCR amplifications used forward primers labeled with M13 tails (5′-TGTAAAACGACGGCCAGT-3′) at the 5′ ends (Schuelke 2000). Amplifications were performed at an 11-µl final volume of 1X PCR Buffer (colorless Flexi GoTaq PCR buffer), 0.2 mM DNTPs, 1.5 mM $MgCl_2$, 0.025 U/µL *Taq* Polymerase (all Promega Corporation, Zurich, Switzerland), 0.18 µg/µL bovine serum albumin (BioConcept, Allschwil, Switzerland), 1 µL DNA, 0.08 µl forward primers with M13 tails, 0.32 µl reverse primers and 0.32 µl M13-primer universal tails labeled with either FAM (Lm123977) or ATTO565 (Lm435135) (Microsynth, Balgach, Switzerland). Touchdown PCRs were carried out on a Bio-Rad Dyad Cycler (Bio-Rad Laboratories, Hercules, California, USA) with the following conditions: 94°C for 5 min; 5X (denaturation 94°C/30 s, starting annealing temperature 62.5°C (Lm123977) or 60.5°C (Lm435135)/45 s, decreasing by 1°C/cycle, extension 72°C/30 s); 20X (denaturation 94°C/30 s, annealing 57.5°C (Lm123977) or 56.5°C (Lm_435135)/45 s, extension 72°C/30 s); 8X (denaturation 94°C/30 s, annealing 53°C/45 s, extension 72°C/30 s); final extension 72°C/10 min and subsequent storage at 10°C.

Text S2. Candidate male-specific contigs obtained from privacy rarefaction, and screened during development of molecular sexing primers.

>ref_PE_reads140_cutoff1_cdhit0.95__E131596_L140+PEcontig1_2114_16953_15949/1

AATTCTTAGCTGTCCTCAGAGAAAATCAAGAAGCCATAGGTTGGACCATGGTAGACATCAAGGGGATTGGCCCCTTAGTCG

TCCAACATCAAAGTCATTTAGGAGAAGAAGCCAAGCCAACTAGAGAACCCTAGAGAAGGCTTATCNNNNNNNNNNGATTC

CCATAGCACCAGAGGACCAGGAGAAGACTACATTCACCTATCCATTTGGAACCTTTGCCTATAGACATATGCCCTTTGGTTT

GTGTAATGCTCCATCTACTTTCTAAAGATGCATGATCAGCATCTTTTCTGATATGATCG

>ref_PE_reads140_cutoff1_cdhit0.95__E131596_L140+PEcontig1_2114_16940_15931/1

AATTCTTAGCTGTCCTCAGAGAAAATCAAGAAGCCATAGGTTGGACCATGGTAGACATCAAGGGGATTGGCCCCTTAGTCG

TCCAACATCAAAGTCATTTAGGAGAAGAAGCCAAGCCAACTAGAGAACCCTAGAGAAGGCTTATCNNNNNNNNNNGATTC

CCATAGCACCAGAGGACCAGGAGAAGACTACATTCACCTATCCATTTGGAACCTTTGCCTATAGACATATGCCCTTTGGTTT

GTGTAATGCTCCATCTCCTTTCTAAAGATGCATGATCAGCATCTTTTCTGATATGATCG

>ref_PE_reads140_cutoff1_cdhit0.95__E131596_L140+PEcontig1_2101_23193_8506/1

AATTCTTAGCTGTCCTCAGAGAAAATCAAGAAGCCATAGGTTGGACCATGGTAGACATCAAGGGGATTGGCCCCTTAGTCG

TCCAACATCAAAGTCATTTAGGAGAAGAAGCCAAGCCAACTAGAGAACCCTAGAGAAGGCTTATCNNNNNNNNNNAATTA

CCATAGCTCCAGAGGAGCAGGAGAAGACTACATTCACCTATCCATTTGGAACCTTTGCCTATAGACATATGCCCTTTGGTTT

GTGTAATGCTCCATCTACTTTCTAAAGAGGCATGATCAGCATCTTTTCTGATATGATCG

>ref_PE_reads140_cutoff1_cdhit0.95__E251152_L140+PEcontig1_2108_16380_20534/1

AATTCAAGACCTTCACATTTTCCCTAGTATTGGAAGCTTCAAGCTCATCTGATTTCTAGCAATTATTACTTAATGTCAAGTTGA

TTTCCTATGTAATTTCTATTTTCCATCAATTTAATTAATGTCTTGTTTTCCTCTGTTGGTTCNNNNNNNNNNCTTCTTCATCCTCT

TCTTCTTCTTCTTCTTCTTCTTCTTCGTCCTCTTCTTCTTCTTCTTCTTCTTCTTCTTCTTCTTCTTCTTCCTCTTCTTCCTCTTCCTC

T

TCTTCTTCTTCTTTCTCTTCTTCTTTTTCTTCTTCCTCATCG

>ref_PE_reads140_cutoff1_cdhit0.95__E251152_L140+PEcontig1_1107_12484_7077/1

AATTCAAGACCTTCACATTTTCCCTAGTATTGGAAGCTTCAAGCTCATCTGATTTCTAGCAATTATTACTTAATGTCAAGTTGA

T

TTCCTATGTAATTTCTATTTTCCATCAATTTAATTAATGTCTTGTTTTCCTCTGTTGGTTCNNNNNNNNNNCATCTTCTTCATCCA

CTTCTTCTTCTTCTTCTTCTTCTTCTTCTTCCTCTTCTTCTTCTTCTTCTTCTTCTTCTTCTTCTTCTTCCTCTTCTTCCTCTTCCTCT

T

CTTCTTCTTCTTTCTCTTCTTCTTTTTCTTCTTCCTCATCG

>ref_PE_reads140_cutoff1_cdhit0.95__E251152_L140+PEcontig1_1101_16968_20192/1

AATTCAAGACCTTCACATTTTCCCTAGTATTGGAAGCTTCAAGCTCATCTGATTTCTAGCAATTATTACTTAATGTCAAGTTGA

T

TTCCTATGTAATTTCTATTTTCCATCAATTTAATTAATGTCTTGTTTTCCTCTGTTGGTTCNNNNNNNNNNCTTCTTCTTCTT

C

TTCTTCTTCTTCTTCTTCTTCTTCTTCCTCTTCTTCTTCTTCTTCTTCTTCTTCTTCTTCTTCTTCTTCCTCTTCTTCCTCTTCCTCTT

C

TTCTTCTTCTTTCTCTTCTTCTTTTTCTTCTTCCTCATCG

>ref_PE_reads140_cutoff1_cdhit0.95__E62062_L140

AATTCCTAAAAAAATACTTCCCCATAGGAAAAACTAATCAACTTAGAAAAGTCATAACTAGTTTTTCCCAGATGGATGGGG

AGATGTTTCATAAGAGCTGGGAGAGGATGAAAGAACTTATTAGGAAATGTCCATATCAT

>ref_PE_reads140_cutoff1_cdhit0.95__E87844_L140+PEcontig1_2102_21849_11167/1

AATTCTATATGCCTCGGCAGTAGGTTCTATCATATATGCCATGACATGTACAAGACTGGATGTGGCCTACTCACTAGGGGT

AGTGAGTAGATACCAGTCTAATCCACGTAAGAACCATTGGAATGTTGTAAAGACAATCCTTAAGTNNNNNNNNNNTGAGC

TTGGAGTGGCACCCTCTATTGATGGTCCAGTTCTTCTGTATTGTGACAGCACTAGAGCAATAGCTCAAGTGAAAGAACCGA

AATCCCATCAGAGAACCAAGCACATTCTAGCTATCACCTTATACGAGAGATCGTAGATCG

>ref_PE_reads146_cutoff1_cdhit0.95__E63057_L207

AATTCTATATGCCTCGGCAGTAGGTTCTATCATATATGCCATGACATGTACAAGACTGGATGTGGCCTACTCACTAGGGGT

AGTGAGTAGATACCAGTCTAATCCACGTAAGAACCATTGGAATGTTGTAAAGACAATCCTTAAGTNNNNNNNNNNTGGAG

TATATTGTGGCATCCGATACTGTAAAGGAAGCTGTTTGGTTGCGAAAGTTCATCAATGAGCTTGGAGTGGCACCCTCTATT

GATGGTCCAGTTCTTCTGTATTGTGACAGCACTAGAGCAATAGCTCAAGTGAAAGAACCGAAATCCCATCAGAGAACCAA

GCACATTCTAGCTATCACCTTATACGAGAGATCGTAGATCG

>ref_SE_cutoff10_vsearch0.8_cdhit0.8__123977_L131+PEcontig1_2103_21536_9724/1

AATTCAAAGCAAAACCACTAAAACCATGGAAACAACCCGCCTAAGGCCGGACCAACAAAATGTGGCCCAAAGATAATTGG

CCCAAGCCCGTTTCCGGCTTGGTCGGGATTGGGTCTCAGTCACAGGCTTTTGGGCTAAAAAAACCTNNNNNNNNNNTTAGC

ATTCACATACTACTGTTGCTACAAAGCTCTCAACTTTTGGTGTGGATCGTAAATGTTTTGTGACTATTTAAGATTAACCGTCA

TTAAATTTAGAACTTTATATGACAGATTTATAGCTACCATTAAATATCTTCATTATCG

>ref_SE_cutoff10_vsearch0.8_cdhit0.8__141017_L141

AATTCTAAAACGAAAGCCTCTAGAGTCCTAAAGCTAAGAGAGATAGATGAGAAGATTTATATACATACATACATACATAC

ATACATACATACATACATACATACATATATATATATATATATATATATTGGATTGTTAGCAGA

>ref_SE_cutoff10_vsearch0.8_cdhit0.8__32815_L142

AATTCAAGACCTTCACATTTTCAGCTAGTATTGGAAGCTTCAAGCTCATCTGATTTCTAGCAATTATTAATTAATGTCAAGT

TGATTTCCTATGTAATTTCTATTTTCCATCAATTTAATTAATGTCTTTCACGTTTTCCAT

>ref_SE_cutoff10_vsearch0.95_cdhit0.95__112479_L131+PEcontig1_2105_14315_9655/1

AATTCTACTGTACAAATCCGAGATTGAAATAACTTAAATCAAATCTGACCAATGGTTGGTGTCACGTAACTGGTCAAATCA

CGTCCAGTGAGTAACTCATTACTCCCATGAGTCTTAAGTATGCGAAGATCACATATCCACTCAGGNNNNNNNNNNCAAAA

ATATGAGAAGACTATGTTAAGAACGATAGACAGAAATTAAATCAAATAGGCAAAGCAGTCCTAGATTCAATAAAAGTGGA

TCTAAGGTGATTGAGAAATCACCTTTAAATTAGAACAAATCACTCTAGAATTAATGTGTCG

>ref_SE_cutoff10_vsearch0.95_cdhit0.95__112743_L131

AATTCATTTTTCGTCATTAACAATCTCTATTTCCTGTATTTCCAAATTTTGATTTCAAGACTTATGACAACAACTTTTATTTCG

TTGTAATAAATAATTTATTACAATGAATAGTTCGCCATTAAAAATAT

>ref_SE_cutoff10_vsearch0.95_cdhit0.95__11918_L131

AATTCTTTAAAACTTCTTGTTATCAAATTGTCACCCATTATCAGTGACAATGGCTCGGGGAAGTCTAAATCTCCATATAATA

AACTTCTAAATAAAGTCTTTTGTCTTAGCCTCAGTTATCTATGCGACTA

>ref_SE_cutoff10_vsearch0.95_cdhit0.95__131907_L131

AATTCATCATAAGAAATATTTTTAACGACAAATCATTCATTGTAATAAATTGTTTATTATGACAAAATAAAATGCATCGTAA

TAAGTCTTGAAACCAAAATTTAGAAATGTAGGAAATAAAGATTTTTAAT

>ref_SE_cutoff10_vsearch0.95_cdhit0.95__168137_L131

AATTCCATCAAAAAATTTACTGACAAATAATTTATCAGTAAATCAGTTACAAATATTTTTAATTTTTTTTAATTTTCTTTTAAA

AAATTTTTTGATAGATTTTCTGACAGAATTGGTTCTGACAAATAATC

>ref_SE_cutoff10_vsearch0.95_cdhit0.95__252108_L132

AAATCTTTTTTTTTTATTTCTGTAGAGGATTTGATCTGGGGAGAGGGTATACAGGATAGTAGGTAGAACTTTAGGTTGTCGGT

TGACCCCTAAGAAAGAGGCATCTTAGGGGAGAAGTTCTGCTTTCTACAC

>ref_SE_cutoff10_vsearch0.95_cdhit0.95__378715_L131

AATTCATTTTTCGTCATTAAAAATCCCTATTTCTTGCATTTTCAAATTTTAGTTTCAAGACTTATTATGACAAATTTTAATTCAT

CATAATAAACCTTTTATTACGACGAATGATTCATCATGAAAAATAT

>ref_SE_cutoff10_vsearch0.95_cdhit0.95__390604_L131

AATTCATCCTACCATGAAGACGGCCAGAGAGTTCGTCCCACTGTGAAGACGGCCGGGGACTTTATTTTGCCCACCGTGAAG

ATGGTCAGGACTTTCAAGTAGGGCTTGGATTACTTAATAGCAAAGTAGTA

>ref_SE_cutoff10_vsearch0.95_cdhit0.95__391552_L131

AATTCTTTTCTCTTTCTCTTCTCTTCTCTCATCTATCCGTTTCTTACAATAAAAAAAAAAAGTGACTTGGAAAAGGGAATCCG

GACAACTAGTAAAAATTGATCCAAGTCCAGTAGACCTTAAGGACACCA

>ref_SE_cutoff10_vsearch0.95_cdhit0.95__435135_L131

AATTCAAATATCAGCTTCACAAGTATTTTATAGATGTTGGTGGTGAGATGAATGTAAGTGAAGTCAAGCAACATAAGCTT

AATCGTGTCTTCTAAAGTGATTGGAAATATCTTTGTGATTATTTCCATTCA

>ref_SE_cutoff10_vsearch0.95_cdhit0.95__439085_L131

AATTCATTTACCTTGTGGATTTTGTGATCTTAGAGACTGAACCAGTAGCAAACCCTAATGGTCACATCCTAGTCATCCTAC

AAAGACCATTTTTAGCCACCACCAATGCCCTAATCAACTGTCACAATGGA

>ref_SE_cutoff10_vsearch0.95_cdhit0.95__489860_L131

AATTCTTGGCCATCATCTTCGCCTTTGAAAAGTTTAGGTCCTATTTGGTTGGGTCACATATTATTGTGTACACTGATCACTT

AGCCATTAGACACCTCTTGATAAAGAAAGATGCTAAGGCACGATTGATC

>ref_SE_cutoff10_vsearch0.95_cdhit0.95__490544_L131

AATTCATTTTTCTTAGAATCTTTTTATATATTTTTATGATTTAATCTTAGAAATAACTTATGACTCTGAGAAACAGATTGTG

ATCAAGGAATACGGTCTATACTCTAGCAAGATAAGTTATGTTCAAAAAT

>ref_SE_cutoff10_vsearch0.95_cdhit0.95__513384_L131

AATTCAACCGTATATTTTTCTGTTCGTCAATTTTTTCTTTCCGATATTGAGTAATGTATTCGGTTTTTTGCACTTCATTCATG

ATATTCTCATCGTCAAGAAATCCATGGAGAAGCTCAGCGAGCTTAGCC

>ref_SE_cutoff10_vsearch0.95_cdhit0.95__593422_L131

AATTCATCTAAAAATTTATAGCACTTGTGTTTATGATGGCTAACTCAGCATTTGGGTACTAGCTTGTAAAGGTCAATTGTAT

CTTCTCTTATCACCTATTTTGGTTAATTTTCTATACAACCCCCCAAATT

>ref_SE_cutoff10_vsearch0.95_cdhit0.95__61023_L131+PEcontig1_2109_18468_6642/1

AATTCCCTGGCCATCTTCATGAAGAAGCCCTGGCCGTCTTCACGATAGGCAAAATAAAACCATTGTCGTTTTCACGATAGG

ATAATATTTAAATCTGACCTCAGTCAGATACACATAGGAATCCGACCCCAGTCGGGACACTACTTNNNNNNNNNNTTCCG

CCGACTCGGAAATCCACTGAGAATGACACCCACTACACCATCACCTTCAACGGCACCAACGATATCACGACGTGGAGGTG

TGGCTCACCAAGATCCTCCACATCCACCGGTGCCGCCTCTCCCATCTCGTCGTCAGCTTCG

>ref_SE_cutoff10_vsearch0.95_cdhit0.95__61023_L131+PEcontig1_2105_18175_22718/1

AATTCCCTGGCCATCTTCATGAAGAAGCCCTGGCCGTCTTCACGATAGGCAAAATAAAACCATTGTCGTTTTCACGATAG

GATAATATTTAAATCTGACCTCAGTCAGATACACATAGGAATCCGACCCCAGTCGGGACACTACTTNNNNNNNNNNTTC

CGCCGACTCGGAAATCCACTGAGAATGACACCCACTACACCATCACCTACAACGGCACCAACGATATCACGGCGTGGAG

GTGTGGCTCACCAAGATCCTCCCCATCCACCGGTGCCGCCTCTCCCATCTCGTCGTCAGCTTCG

>ref_SE_cutoff10_vsearch0.95_cdhit0.95__72787_L131

AATTCAAGACCTTCACATTTTCTCTAGTATTGGAAGCTTCAAGCTCATCTGATTTCTAGCAATTATTAATTAATGTCAAGT

TGATTTCCTATGTAATTTCTATTTTCCATCAATTTAATTAATGTCTTGTT

>ref_SE_cutoff10_vsearch0.9_cdhit0.9__8995_L131

AATTCTTCAAAACTTCTTGTTATCAAATTATCACCCATTATCAGTGACAATGGCTCAGGGAAGTCTAAATCTCCATATAAT

AGACTTCTAAATGAAGTCTTTTGTCTTAGCCTCAGTTATCTGTGCGACTA

‘N’ represents an unknown number of bases in paired-end contigs.

References

Koressaar T, Remm M. 2007. Enhancements and modifications of primer design program Primer3. *Bioinformatics* 23:1289-1291.

Morgan EJ, Kaiser-Bunbury CN, Edwards PJ, Fleischer-Dogley F, Kettle CJ. 2017b. Keeping it in the family: strong fine-scale spatial genetic structure and inbreeding in *Lodoicea maldivica,* the largest-seeded plant in the world. *Conservation Genetics* 18:1317-1329.

Schuelke M. 2000. An economic method for the fluorescent labelling of PCR fragments. *Nature Biotechnology* 18:233-234.

Untergasser A, Cutcutache I, Koressaar T, Ye J, Faircloth BC, Remm M, Rozen SG. 2012. Primer3–new capabilities and interfaces. *Nucleic Acids Research* 40:e115.
